# Protocol for isolation and culture establishment of adult murine neural stem cells using tissue grinder and dissociator-based mechanical dissociation

**DOI:** 10.1016/j.xpro.2026.104908

**Published:** 2026-10-20

**Authors:** Vanessa Arnold, Anna Wolfram, Anastassiya Moskalchuk, Vijayasarathy Sampath-Kumar, Abhirami Sunil, Sogand Ahari, Lisa Sevenich

**Affiliations:** 1 Department of Neurology and Interdisciplinary Neuro-oncology, Hertie Institute for Clinical Brain Research, University Hospital Tübingen, Tübingen, Germany; 2 M3 Research Center for Malignome, Metabolome and Microbiome, University Hospital Tübingen, Tübingen, Germany; 3 Graduate Training Centre of Neuroscience, University of Tübingen, Tübingen, Germany; 4 Faculty of Pharmacy, University of Castilla-La Mancha, Albacete, Spain; 5 Instituto Regional de Investigación Científica Aplicada (IRICA), University of Castilla-La Mancha, Ciudad Real, Spain; 6 Cluster of Excellence iFIT (EXC 2180) ‘Image-Guided and Functionally Instructed Tumor Therapies’, University of Tübingen, Tübingen, Germany

**Keywords:** Neuroscience, Organoids, Stem Cells

## Abstract

Here, we present an enzyme-free workflow for isolating murine adult neural stem cells (aNSCs) using tissue grinder- and dissociator-based mechanical dissociation (TIGR-MD). We describe dissection of the dentate gyrus and subventricular zone, mechanical tissue dissociation, cell recovery, and establishment of primary neurosphere cultures. We further provide procedures for neurosphere maintenance, passaging, and validation. TIGR-MD avoids enzymatic exposure during initial tissue dissociation and supports standardized establishment of primary murine aNSC cultures.

## Highlights

• Steps for dissection of adult murine neurogenic brain regions
• Procedures for enzyme-free mechanical tissue dissociation using TIGR-MD
• Guidance on primary neurosphere culture establishment and maintenance
• Instructions for adult neurosphere passaging and continued culture

## Abstract (editor highlights)

Publisher’s note: Undertaking any experimental protocol requires adherence to local institutional guidelines for laboratory safety and ethics.

## Graphical abstract

**Figure:**
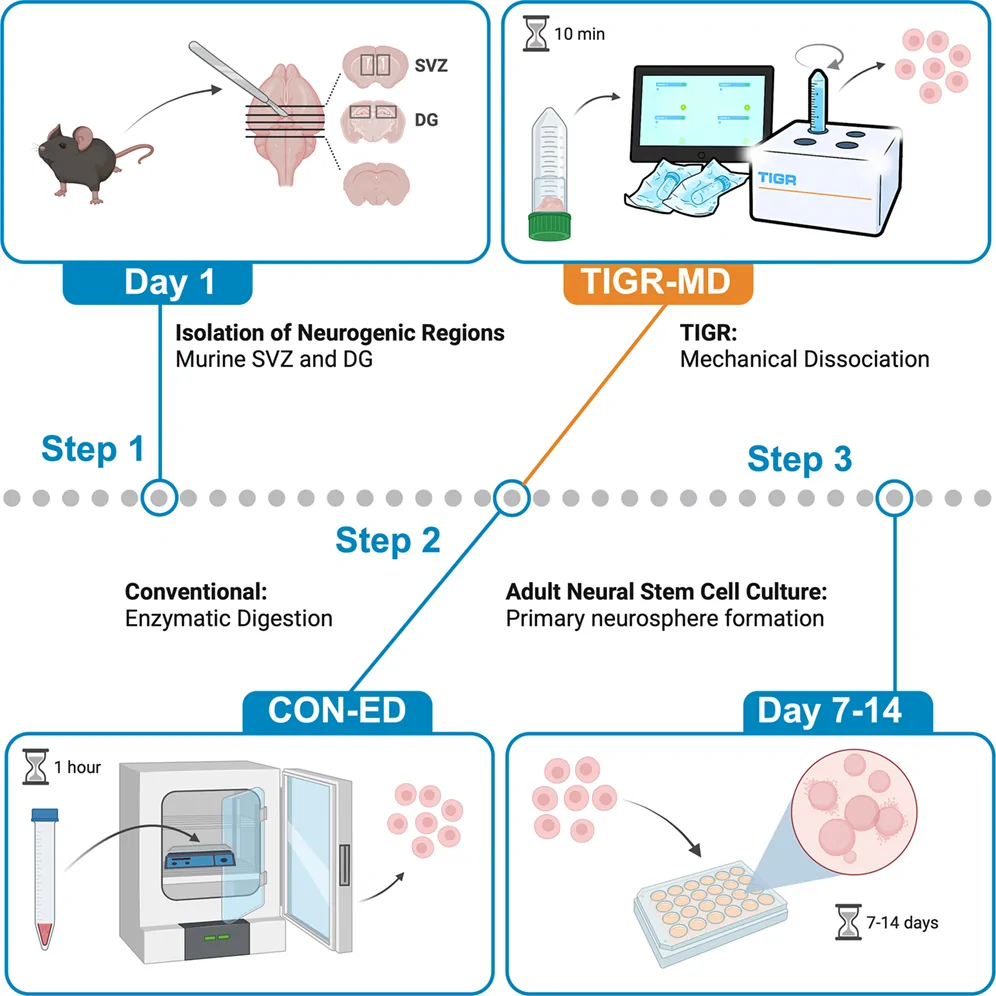

## Before you begin

Murine adult neural stem cells (aNSCs) reside in discrete neurogenic niches of the adult brain, primarily the subventricular zone (SVZ) and the subgranular zone (SGZ) of the hippocampal dentate gyrus (DG). These multipotent cells retain self-renewal capacity and can differentiate into neurons, astrocytes, and oligodendrocytes, supporting adult neurogenesis and contributing to neural plasticity and tissue homeostasis. Murine aNSCs are therefore widely used to study mechanisms of brain health and disease and to develop regenerative strategies for neurological disorders.

In addition, murine aNSCs provide a cellular basis for advanced *ex vivo* models, including three-dimensional (3D) neural cultures and cerebral organoids. These systems enable controlled analysis of cell-cell and cell-matrix interactions, disease mechanisms, and preclinical drug responses.^1^

Conventional murine aNSC isolation methods typically rely on enzymatic digestion of neurogenic tissue using reagents such as trypsin or accutase, here referred to as conventional enzymatic digestion (CON-ED). Although effective, CON-ED can reduce cell viability, alter surface epitopes, and affect the recovery of sensitive cell populations, which may compromise early-stage cultures and downstream applications.

Here, we describe an enzyme-free protocol for isolating murine aNSCs using the Tissue Grinder and Dissociator (TIGR)-based mechanical dissociation (TIGR-MD) with the TIGR Tissue Grinder & Dissociator, which employs a technical system comparable or identical to that used in similar commercially available tissue grinders.

TIGR-MD provides a rapid, enzyme-free alternative to CON-ED and supports the efficient recovery of primary cells and successful establishment of neurosphere cultures. The present protocol focuses specifically on primary cell isolation, neurosphere initiation, and early culture expansion. Here, *enzyme-free* refers specifically to the initial dissociation of primary brain tissue; established neurosphere cultures are subsequently passaged using trypsin-EDTA. The resulting cultures provide a suitable starting population for established downstream differentiation workflows, including 2D and 3D neural *in vitro* and *ex vivo* models.^2^

### Innovation

This protocol introduces a standardized, enzyme-free TIGR-MD workflow for isolating murine adult neural stem cell-enriched cultures from neurogenic brain regions. In contrast to CON-ED, TIGR-MD eliminates enzymatic exposure during the initial tissue dissociation step and reduces active processing time. The key innovation is the use of controlled rotor-stator-based mechanical dissociation instead of enzymatic digestion and extensive manual trituration, thereby reducing handling and operator-dependent variability during primary culture establishment.

Successful initial culture establishment is a particularly critical step because aNSCs are isolated from limited primary tissue obtained from animals. Thus, maximizing cell recovery and reliable neurosphere initiation helps to prevent the loss of valuable biological material and may therefore reduce the need for additional tissue collection. In representative day 12 cultures, TIGR-MD resulted in more than three-fold higher neurosphere formation than CON-ED, supporting its use for efficient early aNSC culture establishment.

The present protocol focuses specifically on primary tissue dissociation, neurosphere initiation, and early culture establishment. Long-term expansion, expression of neural stem cell markers, and differentiation into neuronal, astrocytic, and oligodendrocyte-lineage cells have previously been demonstrated using established adult aNSC culture workflows, including Ahmed et al. (2021).^3^

### Institutional permissions

All animal procedures were performed in accordance with relevant institutional, national, and international guidelines and regulations. Tissue collection was performed under the institutional exemption applicable to organ harvesting and was not part of a separately authorized animal experiment; therefore, no dedicated authorization number was issued. In this protocol, brains were collected from C57BL/6 young adult mice aged 9–20 weeks that became available through ongoing animal work at the local institution. Tissue collection and use were conducted in accordance with local institutional and regulatory requirements.

***Note:*** Before applying this protocol, users must obtain approval from the relevant institutional animal care and use committee or equivalent regulatory authority and ensure compliance with all applicable local, national, and institutional regulations.

## Key resources table

**Table:** 

REAGENT or RESOURCE	SOURCE	IDENTIFIER
**Antibodies**		

Goat polyclonal antibody anti-GFAP (1:500)	Abcam	Ab53554
Goat polyclonal antibody anti-SOX2 (1:500)	R&D systems	AF2018-SP
Mouse monoclonal antibody anti-Nestin, clone rat-401 (1:500)	Sigma Aldrich	MAB353
Chicken polyclonal antibody anti-β3-TUBB3 (1:500)	Synaptic Systems	302 306
Alexa Fluor 488 AffiniPure Donkey anti-Chicken IgY (IgG) (H+L), polyclonal (1:500)	Jackson ImmunoResearch	703–545–155
Alexa Fluor® 488-AffiniPure Alpaca Anti-Mouse IgG (H+L) (min X Bov,Hu,Rb Sr Prot)	Jackson ImmunoResearch	615–545–214
Alexa Fluor 647 AffiniPure Bovine anti-Goat IgG (H+L), polyclonal (1:500)	Jackson ImmunoResearch	805–605–180

**Biological samples**		

Mouse adult neural stem cells BL6 WT		

**Chemicals, peptides, and recombinant proteins**		

Recombinant human Fibroblast Growth Factor (FGF) basic	PeproTech	100-18B
Recombinant murine Epidermal Growth Factor (EGF)	PeproTech	315–09
**4′,6-Diamidino-2-phenylindole (DAPI) (1:2000)**	Sigma-Aldrich	D9542
Anti-adherence rinsing solution	Stemcell	07010
B-27™ supplement (50×), minus vitamin A	Gibco	12587010
Antibiotic antimycotic solution (100×), stabilized	Sigma-Aldrich	A5955
Advanced DMEM/F12	Gibco	12634010
GlutaMAX (35050061, Gibco) supplement	Gibco	35050061
HEPES solution	Sigma-Aldrich	H0887
Ethanol, Absolute (200 Proof), Molecular Biology Grade	Fisher Scientific	10048291
Trypan blue	Sigma-Aldrich	T8154

**Software and algorithms**		

Prism 11 Version 11.0.2 (100)	GraphPad Prism	N/A
TIGR Software Version 1_0_9	OMNI Life Science GmbH & Co KG	N/A

**Other**		

CERO 3D Incubator & Bioreactor	OMNI Life Science GmbH & Co KG	N/A
CO_2_ resistant shaker (with 19 mm orbit)	Thermo Fisher Scientific Inc.	88881101
TIGR Tissue Grinder & Dissociator	OMNI Life Science GmbH & Co KG	990700
24-well ultra-low attachment plates	Thermo Fisher Scientific	174930
Bonn Scissors	Fine Science Tools	14084–08
Eppendorf conical tube (5 mL)	Eppendorf	EP0030122321-200EA
Filter tips (10 μL)	Star lab	S1121-3810
Filter tips (20 μL)	Biozayn	25158
Filter tips (200 μL)	Green-tip	06–662–5300
Filter tips (1000 μL)	Green-tip	06–695–5300
Microtubes (1.5 mL)	Sarstedt	72.706
Hemocytometer counting chamber	KOVA Glasstic	87144E
Pipettes (5 mL)	Integra	20270916
Falcon tubes 15 mL	Greiner Bio-One	188271
Falcon tubes 50 mL	Greiner	227261
Hank’s Buffered Salt Solution (HBSS)	Gibco	14025–126
MACS SmartStrainer 100 μm	Miltenyi Biotec	130–098–463
Neural stem cell basal medium	EMD Millipore	SCM003
Petri dish	VWR	391–0561
Pipettes (25 mL)	Integra	20270909
Scalpel	VWR	233–0034
Tissue Culture Flask, treated, vented cap, 50 mL volume, surface 25,0 cm2	OMNI Life Science GmbH & Co KG	TCG4
Trypsin inhibitor	Sigma-Aldrich	T6522
Trypsin-EDTA (0.05%)	Gibco	25300054
TIGR Tubes	OMNI Life Science GmbH & Co KG	990706

## Materials and equipment

**Table:** aNSC dissection buffer

Reagent	Final concentration	Amount
Hank’s Buffered Salt Solution (HBSS)	1×	50 mL
4-(2-Hydroxyethyl)-1-piperazineethanesulfonic acid (HEPES)	2 mM	23.83 mg
Total	N/A	50 mL

***Note:*** Store at 4°C for up to 2 weeks.

**Table:** Neurosphere wash medium

Reagent	Final concentration	Amount
Advanced DMEM/F12	1×	48 mL
B27 without vitamin A	1×	1 mL
GlutaMAX	1×	500 μL
Antibiotic-Antimycotic	1×	500 μL
Total	N/A	50 mL

***Note:*** Store at 4°C for up to 2 weeks.

**Table:** Neurosphere growth medium

Reagent	Final concentration	Amount
Neural stem cell basal medium	1×	47.97 mL
B27 without vitamin A	1×	1 mL
GlutaMAX	1×	500 μL
Antibiotic-Antimycotic	1×	500 μL
Epidermal growth factor (EGF)	30 ng/mL	15 μL of 100 μg/mL stock per 50 mL
Basic fibroblast growth factor (bFGF)	30 ng/mL	15 μL of 100 μg/mL stock per 50 mL
Total	N/A	50 mL

***Note:*** Prepare fresh for each experiment.

## Step-by-step method details

### Dissection and collection of neural stem cell-rich regions from adult murine brain

**Timing: 30–45 min**

This step describes the dissection of adult mouse brain and the isolation of neural stem cell-rich regions, specifically the dentate gyrus (DG) and subventricular zone (SVZ). Proper identification and clean dissection of these areas are essential for downstream murine aNSC culture.

1. Obtain mouse heads in accordance with approved institutional procedures and all applicable animal welfare regulations.
2. Sterilize the dissection tools, bench area, and mouse heads with 70% ethanol before brain extraction.
3. Surgically remove the brain and immediately transfer it into a 50 mL conical tube containing ice-cold dissection buffer.
4. In a laminar flow hood, slice the brain coronally as shown in Figure 1.

**Figure 1:**
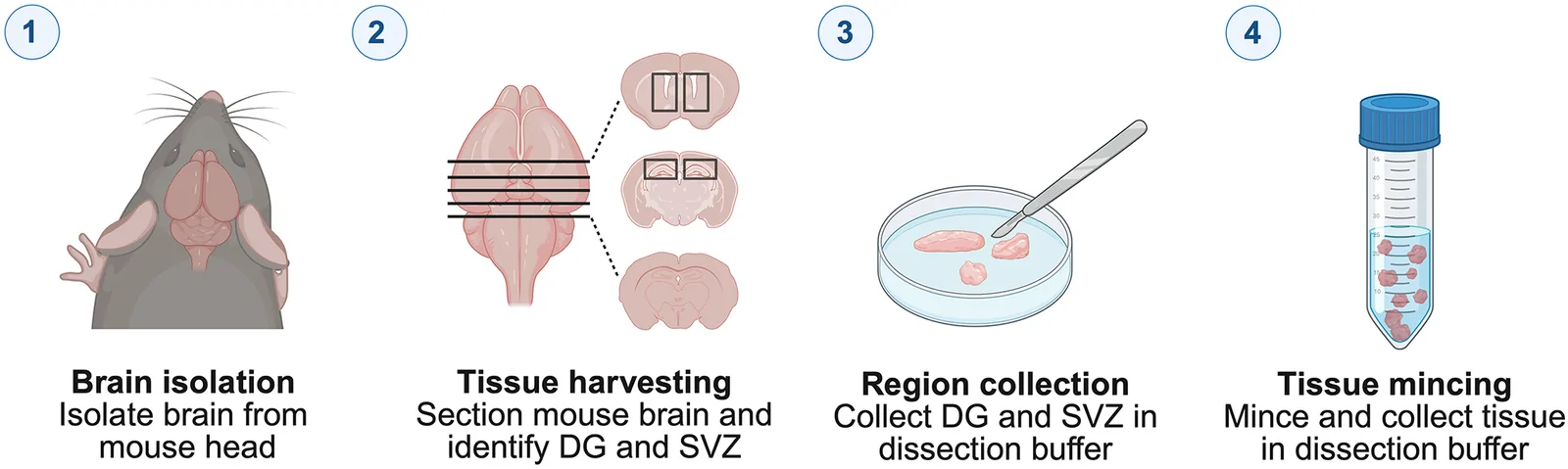
Dissection and collection of neural stem cell-rich regions from adult mouse brain The schematic summarizes the initial tissue preparation workflow for adult murine neural stem cell culture. Following brain isolation from the mouse head, the adult mouse brain is sectioned to identify neural stem cell-rich regions, including the dentate gyrus (DG) and subventricular zone (SVZ). The DG and SVZ are collected in dissection buffer, minced into small tissue fragments, and kept in dissection buffer for subsequent dissociation by either CON-ED or TIGR-MD. Created with BioRender.com.
5. Transfer brain slices containing the DG and SVZ to a petri dish with ice-cold dissection buffer.
6. Isolate the DG and SVZ regions using a scalpel. Mince the tissue with fine scissors in the lid of a 15 mL Falcon tube.
7. Suspend the minced tissue in ice-cold dissection buffer by inverting the tube gently. Keep the tube on ice and add more dissected tissue if processing multiple brains.

***Note:*** For each mouse, the dissected DG and SVZ tissues were pooled before dissociation and processed as a single sample. All validation images and outcome measurements reported in this protocol were obtained from these pooled cultures. Separate region-specific cultures were not established or independently evaluated.

### Tissue dissociation strategies for neural stem cell-rich regions

**Timing: 1 h (for step 8)**

**Timing: 10 min (for step 9)**

Following dissection, neural stem cell-rich tissue can be dissociated using either CON-ED or TIGR-MD. CON-ED represents the conventional workflow commonly used for murine aNSC isolation and is adapted from a previously published protocol by Ahmed *et. al.*, in 2021.^3^ TIGR-MD provides an enzyme-free alternative designed to reduce processing time and preserve cell viability. The two workflows are described below as independent dissociation strategies. Proceed with either CON-ED or TIGR-MD depending on the experimental objective.

8. Part 1: CON-ED – conventional enzymatic digestion for single-cell suspension.
  ***Note:*** The CON-ED workflow outlines an enzyme-based tissue digestion protocol adapted from Ahmed et al. and is included as a reference workflow for comparison with the enzyme-free TIGR-based mechanical dissociation approach.^3^ This workflow uses enzymatic digestion with trypsin-EDTA to dissociate the dissected DG and SVZ tissue into a single-cell suspension suitable for downstream culture. Proper control of digestion time is critical to maintain cell viability and minimize over-digestion (Figure 2).
  a. Centrifuge the minced tissue at 200 × *g* for 5 min at 24°C.
  b. Carefully aspirate the supernatant and add 1 mL of 0.05% trypsin-EDTA.
  c. Incubate the sample at 37°C with gentle orbital shaking for 15–20 min.
    **CRITICAL:** Monitor the tissue during incubation and use the shortest digestion time required to obtain an adequate cell suspension. Early over-digestion may not be visibly apparent but can result in excessive cleavage of cell-surface and adhesion molecules, thereby reducing neurosphere-forming capacity. More prolonged digestion may lead to measurable loss of cell viability. Total digestion time should not exceed 30 min.
  d. Gently pipette the tissue with a P1000 to assess the degree of dissociation. If necessary, continue incubation for an additional 5–15 min.
  e. Add 1 mL of trypsin inhibitor (1 mg/mL) to neutralize the enzyme activity.
    ***Note:*** The stated timing and reagent volumes apply to pooled DG and SVZ tissue from one adult mouse. When processing tissue from additional mice in the same preparation, increase the trypsin-EDTA volume by approximately 0.5 mL per additional brain while ensuring that the tissue remains fully immersed. This protocol was specifically established using tissue from one mouse.
  f. Incubate again at 37°C with orbital shaking for 20 min.
  g. Triturate the tissue by pipetting it up and down 10–20 times. Start with a P1000 to break up larger clumps, then switch to a P200 to achieve a finer suspension. A few small clumps may remain.
    ***Note:*** The number of pipetting cycles may vary depending on tissue consistency, pipetting speed and force, and operator experience. Begin with 10 cycles and assess the suspension. Continue up to 20 cycles only if larger clumps remain. Avoid vigorous or excessive trituration, as this may cause mechanical damage and reduce cell viability.
9. Part 2: TIGR-MD – enzyme-free mechanical tissue dissociation.
  ***Note:*** This enzyme-free approach uses the TIGR Tissue Grinder & Dissociator for rapid mechanical dissociation (Figure 3). The sterile disposable 50 mL TIGR tube contains an integrated rotor–stator grinding unit and a 100 μm cell strainer, allowing tissue dissociation and removal of larger tissue fragments within the same closed assembly. The stator remains stationary while the rotor moves bidirectionally, generating controlled shearing and milling forces.
  a. Transfer dissected tissue into a sterile TIGR tube within a biosafety hood.
    i. Position tissue between the rotor and stator elements of the grinding unit.
    ii. Add approximately 1 mL of wash medium.
    iii. Close the TIGR tube securely and place it into the instrument according to the manufacturer's operating instructions supplied with the TIGR Tissue Grinder & Dissociator.
    iv. Select the manufacturer-provided “Soft” dissociation program and start the dissociation cycle. The Soft protocol consists of six sequential bidirectional rotation steps with a total programmed duration of 100 s:
      Step 1: Rotate at +8 rpm for 15 s with an acceleration time of 5 s.
      Step 2: Rotate at −8 rpm for 15 s with an acceleration time of 5 s.
      Step 3: Rotate at +15 rpm for 20 s with an acceleration time of 5 s.
      Step 4: Rotate at −15 rpm for 20 s with an acceleration time of 5 s.
      Step 5: Rotate at +10 rpm for 15 s with an acceleration time of 5 s.
      Step 6: Rotate at −10 rpm for 15 s with an acceleration time of 5 s.
      ***Note:*** Positive rpm values indicate clockwise rotation, whereas negative values indicate counterclockwise rotation. The 5-s acceleration period is included within the stated duration of each rotational step. Therefore, the total programmed duration of the six-step “Soft” protocol is 100 s.
      ***Note:*** The Soft-protocol parameters cannot be modified through the standard TIGR software interface. The underlying protocol file is stored in JSON format and can, in principle, be edited using a text editor and saved under a new protocol name. The present protocol was established using the unmodified manufacturer provided “Soft” protocol. Any modification of these parameters requires independent optimization and validation.
    v. After completion of the cycle, return the tube to the upright position and allow the dissociated cell suspension to pass through the integrated 100 μm cell strainer into the collection compartment.
      ***Note:*** The TIGR tube combines a rotor–stator grinding unit, a 100 μm cell strainer, and a collection compartment within a sterile 50 mL tube assembly. Bidirectional rotor movement applies controlled mechanical forces to dissociate the tissue, while the cell strainer retains larger undissociated fragments. Consequently, an additional filtration step is not required.
      ***Note:*** Transfer the pooled DG and SVZ tissue from one adult mouse into one TIGR tube. As these regions are small after microdissection, they can be transferred directly from the brain slices without further weighing or extensive mincing. This protocol was established using tissue from one adult mouse per tube; higher tissue loads were not evaluated.
  b. Place the TIGR tube containing the dissociated cell suspension directly into the centrifuge and centrifuge at 200 × *g* for 3 min at 24°C.
    ***Note:*** For TIGR-MD, the integrated strainer within the TIGR tube removes larger tissue fragments during dissociation; therefore, additional filtration is not required.
  c. Carefully discard the supernatant without disturbing the cell pellet.
  d. Gently resuspend the cell pellet in 1 mL of aNSC growth medium.

**Figure 2:**
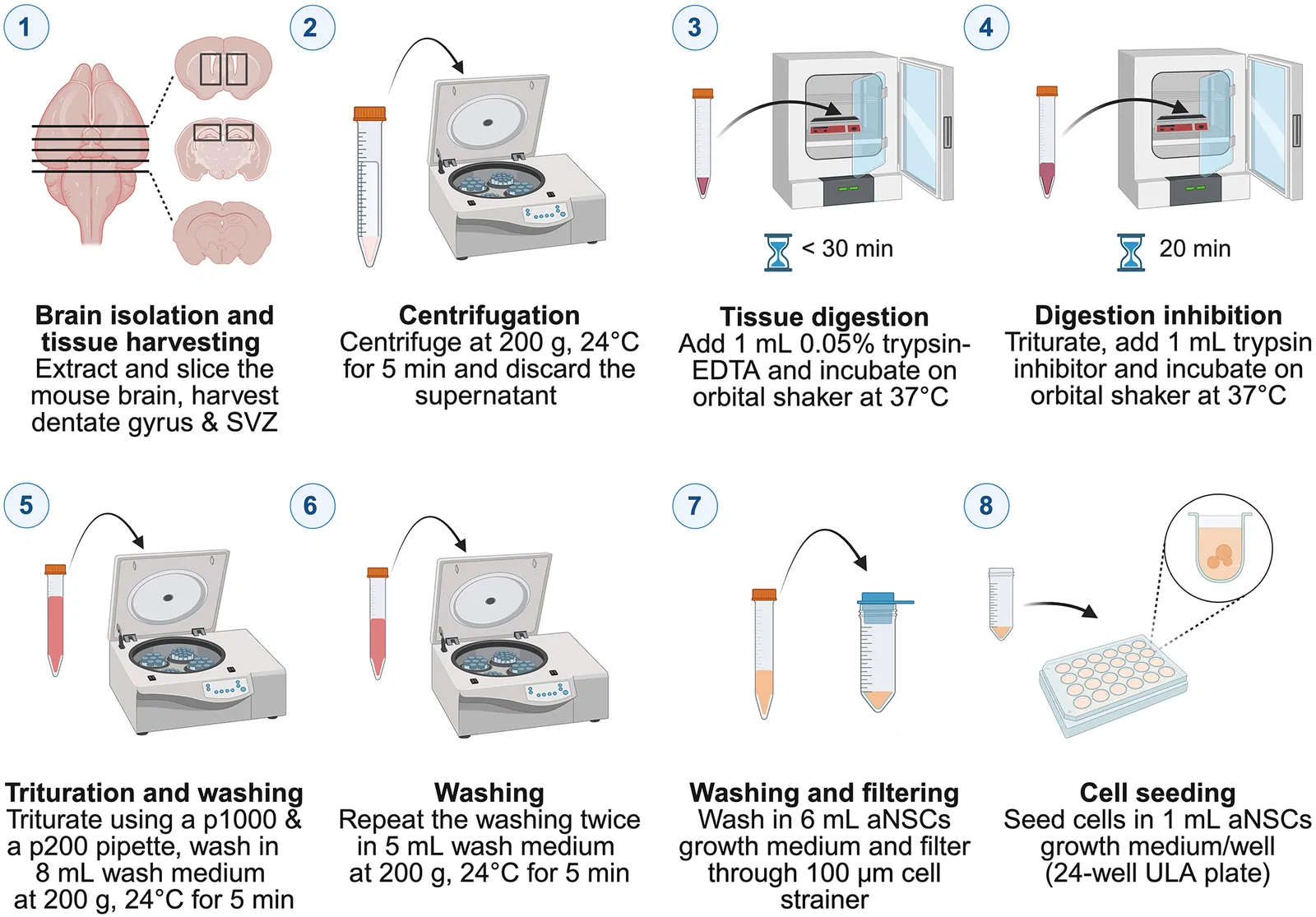
CON-ED workflow for adult murine neural stem cell culture This schematic illustrates the CON-ED workflow used to generate adult neural stem cell cultures from murine neurogenic brain regions. After isolation of the DG and SVZ, the tissue is collected by centrifugation and incubated with 0.05% trypsin-EDTA at 37°C to promote tissue dissociation. Enzymatic activity is then stopped using trypsin inhibitor, followed by careful trituration to further disperse the tissue into a cell suspension. After repeated washing steps and filtration through a 100 μm cell strainer, the recovered cells are transferred into adult neural stem cell growth medium and seeded into a 24-well plate to initiate neurosphere formation. Created with BioRender.com.

**Figure 3:**
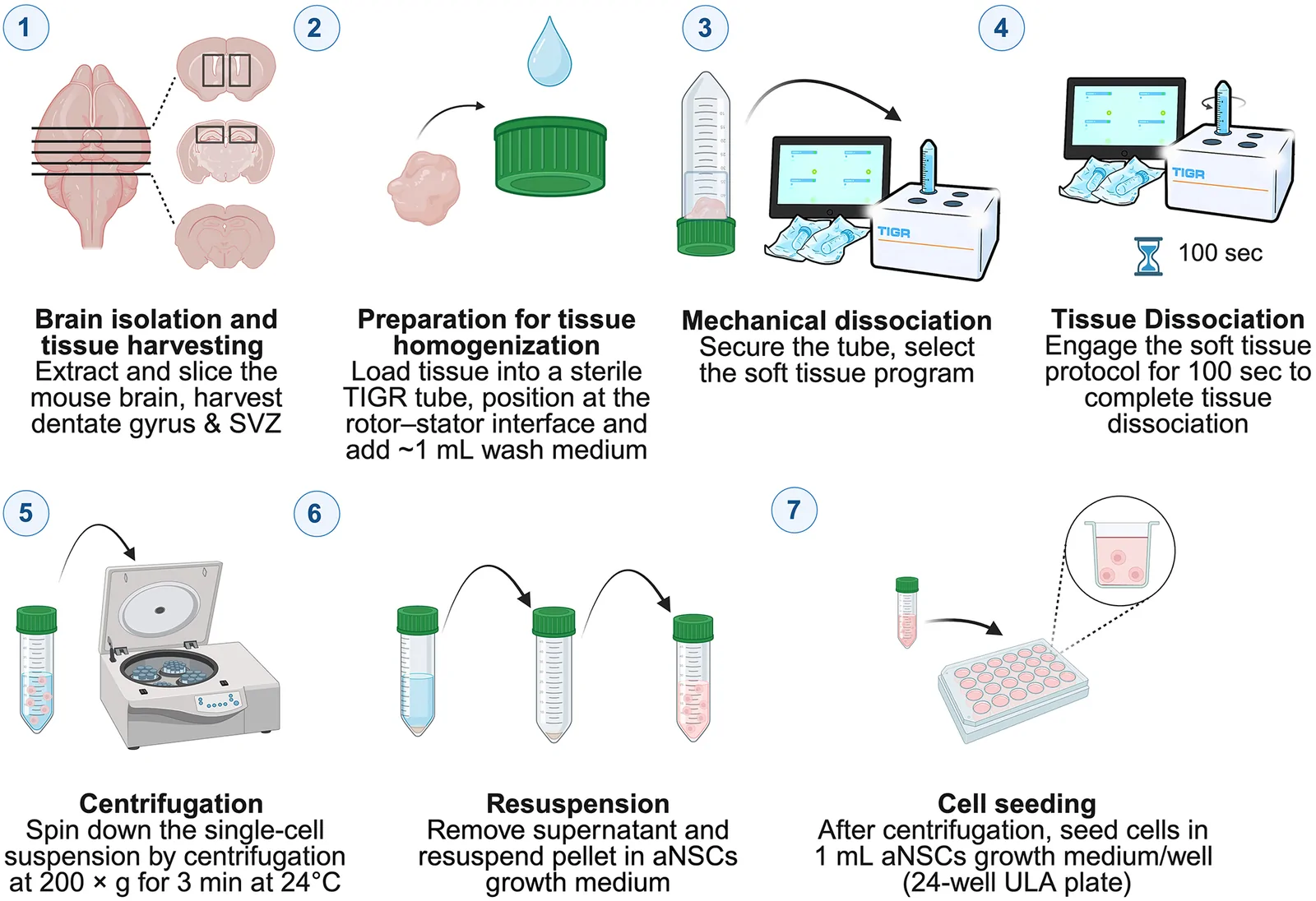
Overview of the TIGR-MD workflow for adult murine neural stem cell culture Adult mouse brains are isolated and sectioned to harvest neural stem cell-rich regions, including the DG and the SVZ. Dissected tissue is transferred into a sterile TIGR tube, positioned at the rotor-stator interface, and supplemented with wash medium. TIGR-MD is performed using the manufacturer-provided “Soft” dissociation program for 100 s. The resulting cell suspension is pelleted by centrifugation, the supernatant is discarded, and the pellet is gently resuspended in aNSC growth medium. Cells are then seeded in a 24-well ultra-low attachment (ULA) plate for neurosphere culture. Created with BioRender.com.

### Cell washing, seeding, and initial culture

**Timing: Approximately 48 h, including approximately 1 h of active handling for CON-ED or 10 min for TIGR-MD**

This step describes cell washing, seeding, and initial culture after tissue dissociation. Washing and filtration are required for CON-ED to remove residual enzyme, inhibitor, and tissue debris. For TIGR-MD, these steps are optional and can be omitted if the cell suspension is sufficiently homogeneous.

10. For CON-ED, or for TIGR-MD if visible debris or small tissue fragments are present, dilute the dissociated cell suspension with wash medium and centrifuge at 300 × *g* for 3–5 min at 24°C.

***Note:*** For TIGR-MD, proceed directly to step 12 unless visible debris or small tissue fragments are present.

11. Aspirate the supernatant and gently resuspend the pellet in fresh wash medium. Repeat the wash step two more times.
  a. After each centrifugation, discard the supernatant and resuspend in wash medium.
  b. After the final centrifugation, resuspend the pellet in aNSC growth medium.
  c. Pass the suspension through a 100 μm cell strainer to remove residual clumps.
12. Seed the entire suspension obtained from the pooled DG and SVZ tissue of one adult mouse into a single well of a 24-well ultra-low attachment (ULA) plate.

***Note:*** Cell counting is not recommended during initial culture establishment, as minimizing handling time and cell loss is critical. Cell yield may vary between preparations; therefore, the seeding input is standardized by tissue source rather than by a predefined cell number.

***Note:*** ULA plates promote neurosphere formation by preventing premature cell adhesion and differentiation.

13. Incubate cultures at 37°C in a humidified incubator with 5% CO_2_ for 48 h.
14. After 48 h, replace half the medium with fresh aNSC growth medium.
  a. Gently aspirate from the well edge to avoid disturbing neurospheres.
  b. Continue half-medium changes daily or every other day until use, depending on cell density and metabolic activity. Cultures with higher cell numbers generally require daily half-medium changes, whereas lower-density cultures can undergo half-medium changes every other day. Medium color should be monitored as an additional indicator, as a shift toward yellow suggests increased acidification and the need for more frequent medium exchange.

***Note:*** For users who are unfamiliar with the culture system, daily half-medium changes are recommended until sufficient experience has been gained to adjust the feeding schedule based on cell density, neurosphere growth, and medium color.

### Adult neurosphere culture and passaging

**Timing: 1–2 h for passaging; 3–5 days between passages**

This section describes the collection and enzymatic dissociation of established neurospheres into a single-cell suspension, followed by reseeding for continued culture expansion. Although TIGR-MD is enzyme-free during the initial dissociation of primary brain tissue and culture establishment, subsequent passaging is performed using brief trypsin-EDTA treatment. Trypsin-EDTA was selected as a cost-effective and routinely available method for dissociating expanded cultures, whereas repeated use of TIGR tubes during passaging would substantially increase consumable costs. Alternative reagents such as accutase were not evaluated in this protocol.

During primary culture establishment, the complete recovered cell suspension is seeded without prior counting because it contains a heterogeneous mixture of proliferative and non-proliferative cells, small aggregates, and tissue debris. During subsequent passages, non-proliferative cells and debris are progressively depleted, allowing established cultures to be counted and reseeded at a standardized viable-cell density.

15. Once neurospheres reach 50–100 μm in diameter, proceed with passaging.
16. Use a P1000 pipette to collect neurospheres into a sterile 5 mL conical tube.
  a. Centrifuge at 300 × *g* for 5 min at 24°C. Aspirate the supernatant.
17. Add 1 mL of 0.05% trypsin-EDTA to the pellet.
  a. Incubate at 37°C for 2 min with gentle agitation.
18. Add 1 mL of trypsin inhibitor (1 mg/mL) to stop digestion.
  a. Begin trituration using a P1000 pipette.
  b. Switch to a P200 pipette to complete single-cell dissociation.

**CRITICAL:** Avoid excessive trituration to preserve cell integrity and viability.

19. Centrifuge the dissociated cells at 300 × *g* for 5 min at 24°C and carefully aspirate the supernatant.
20. Resuspend the pellet in 1–3.5 mL of aNSC growth medium.
21. Seed established cultures at approximately 5 × 10^5^ viable cells per well in a 24-well ULA plate.
  a. Adjust the suspension to 5 × 10^5^ viable cells/mL and add 1 mL per well.
  b. For a T25 flask pre-coated with anti-adherence rinsing solution, use the same viable-cell concentration and add 2 mL of cell suspension.

**CRITICAL:** The density of 5 × 10^5^ viable cells per well is the routine reseeding target for established cultures and supports rapid neurosphere reformation. It does not apply to primary culture establishment, during which the complete recovered suspension is seeded without prior counting.

22. Incubate at 37°C in a humidified incubator with 5% CO_2_ until the next passage (typically 3–5 days).

### Differentiation and immunofluorescence validation of established aNSC cultures

**Timing: 6–7 days**

Differentiation and subsequent immunofluorescence staining of established aNSC cultures for neural stem/progenitor and differentiation markers were performed without modification according to the previously published protocol by Ahmed et al. (2021).^3^ Cultures at passage 6 were used for the experiments shown in Figures 5 and 6. The experiments were performed using **three independent biological replicates per condition**, with each biological replicate representing a culture established from one mouse.

## Expected outcomes

All expected outcomes described below refer to cultures established from pooled DG and SVZ tissue obtained from one adult mouse. Separate region-specific cultures were not established or independently evaluated.

Following successful isolation and seeding, the culture initially contains a heterogeneous mixture of single cells, small cellular aggregates, tissue debris, and surviving neural stem/progenitor-enriched cells. During the first 24–48 h, most cells remain dispersed or form small aggregates. Early spherical structures may become visible within approximately 4–6 days and subsequently develop into compact, free-floating neurospheres. Under successful culture conditions, neurospheres with smooth, phase-bright borders typically reach approximately 50–100 μm in diameter within 7–14 days.

TIGR-MD provides a rapid and standardized workflow for initial aNSC culture establishment while avoiding enzymatic exposure during primary tissue dissociation, TIGR-MD cultures contained 17.0 ± 3.85 neurospheres per predefined 4× field of view, whereas CON-ED cultures contained 5.5 ± 1.38 neurospheres per field of view, corresponding to an approximately 3.1-fold higher neurosphere count under the conditions tested. The progressive development of neurospheres in cultures established using both approaches is shown in Figure 4.

**Figure 4:**
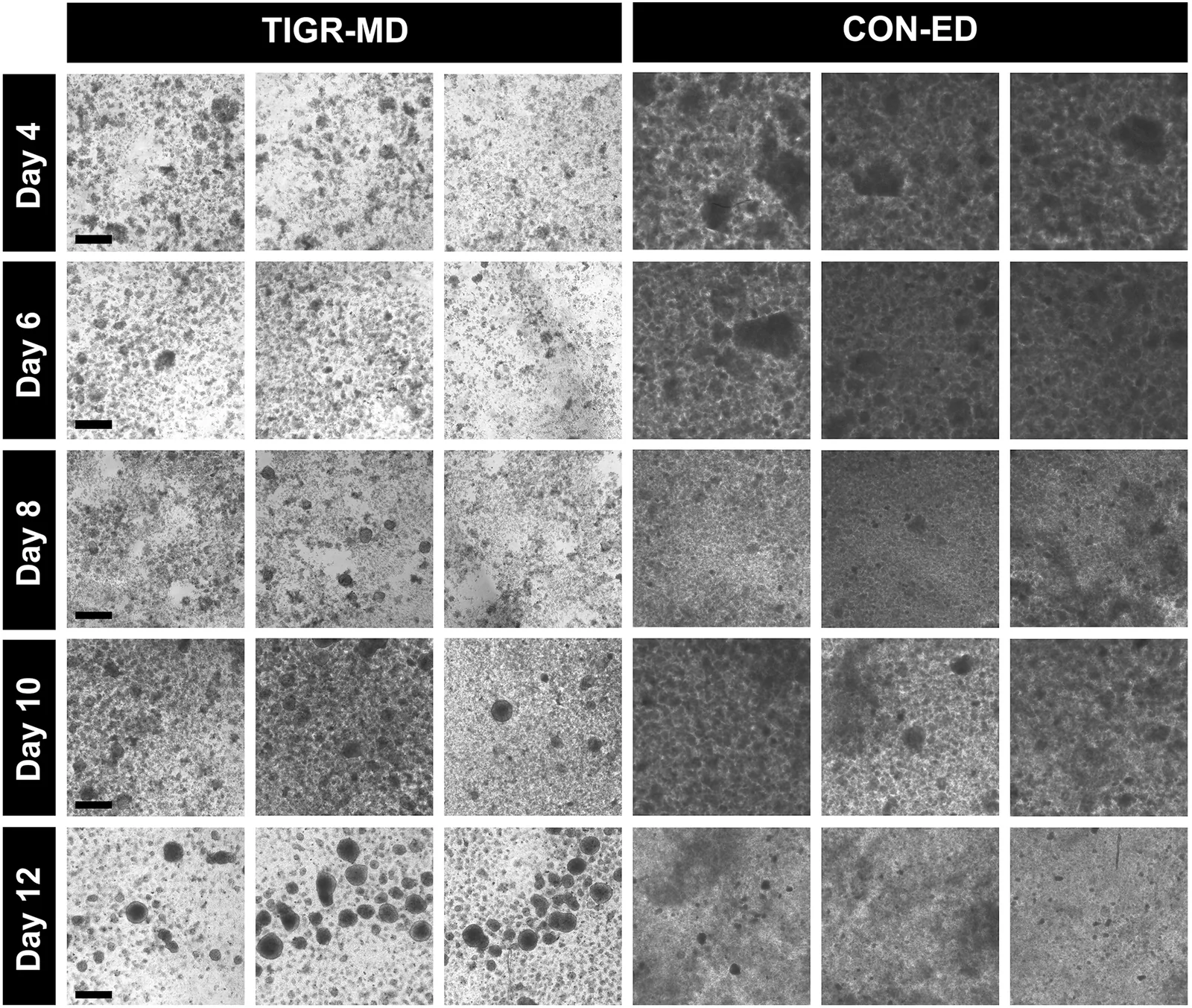
Adult neural stem cell culture following TIGR-MD or CON-ED Representative bright-field images of adult neural stem cell cultures generated using TIGR-MD, shown in the left panel, or CON-ED, shown in the right panel. Cultures were monitored for 12 days after isolation, with representative images shown on days 4, 6, 8, 10, and 12. Representative images show cultures established from pooled DG and SVZ tissue. Under the conditions shown, TIGR-MD cultures exhibit progressive formation of neurospheres, whereas CON-ED cultures show comparatively limited cell aggregation. Images are representative of three independent isolations. Scale bar: 500 μm.

CON-ED can also generate neurosphere cultures but requires careful control of digestion time and trituration intensity. Excessive enzymatic exposure or mechanical stress may reduce viable cell recovery and impair subsequent neurosphere formation. Culture outcomes following CON-ED may therefore be more sensitive to tissue-processing time and operator-dependent handling.

Primary neurospheres should be passaged when they reach approximately 50–100 μm in diameter. Following dissociation, the culture should contain predominantly single cells and small aggregates, which should reform neurospheres within 3–5 days. Representative passage-10 cultures established using both TIGR-MD and CON-ED showed successful neurosphere maintenance and recovery following splitting.

At passage 10, TIGR-MD-derived cultures showed a higher live-cell concentration and a lower proportion of dead cells after splitting than CON-ED-derived cultures, indicating improved recovery and culture quality during continued expansion under the conditions tested (Figure 9). For reseeding established cultures after passaging, seed approximately 5 × 10^5^ viable cells per well in 1 mL of aNSC growth medium in a 24-well ULA plate. This density represents the routine target for rapid neurosphere reformation. During primary culture establishment, the entire recovered cell suspension from one mouse is seeded without prior counting.

In our hands, cultures established using both dissociation approaches were maintained for at least 20 passages and successfully recovered following repeated freezing and thawing. Formal assessment of clonal self-renewal, however, requires dedicated secondary neurosphere formation assays.

Culture quality can be assessed by the presence of round, non-adherent neurospheres with limited debris accumulation and continued proliferation in growth factor-supplemented medium. Healthy cultures should remain predominantly in suspension in ULA vessels and display consistent neurosphere growth between passages. In contrast, irregularly shaped or dark neurospheres, extensive debris, substantial cell attachment, absence of sphere growth, or poor recovery after passaging may indicate over-processing, reduced viability, contamination, or insufficient growth factor activity.

Cultures generated using both TIGR-MD and CON-ED expressed the neural stem cell-associated markers Nestin and SOX2, supporting maintenance of a neural stem/progenitor-associated phenotype following neurosphere establishment (Figure 5). Cells derived from both conditions also differentiated into TUBB3-positive neuronal and GFAP-positive astrocytic lineages under the conditions tested (Figure 6). The resulting cultures therefore provide suitable starting material for established downstream workflows, including immunocytochemistry, molecular profiling, and differentiation assays. Application-specific validation should be performed before their use in further functional studies.

**Figure 5:**
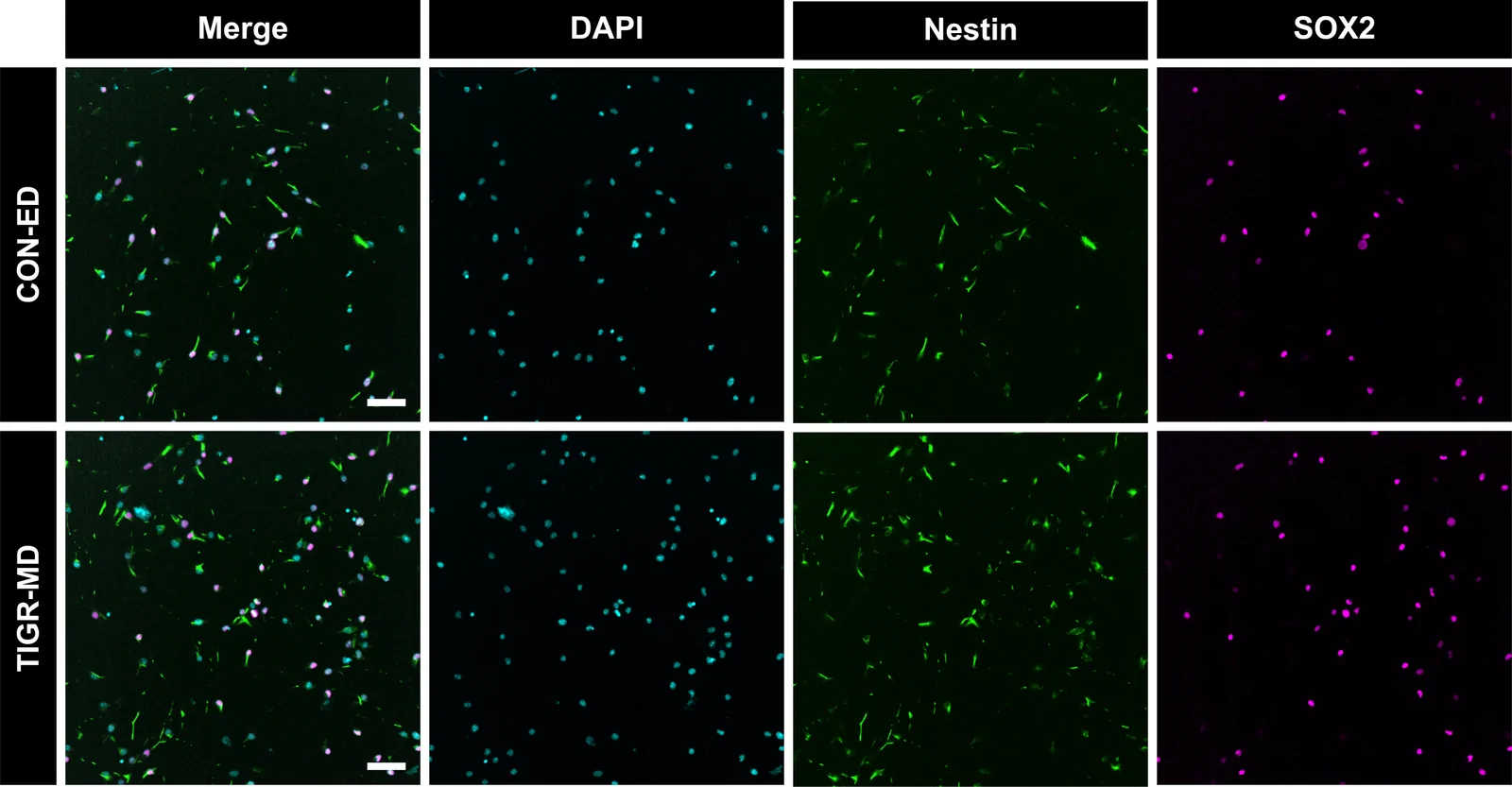
Immunofluorescence validation of neural stem cell marker expression in murine adult neural stem cell cultures Representative immunofluorescence images of murine adult neural stem cell (aNSC) cultures established using CON-ED or TIGR-MD, showing expression of the neural stem cell-associated markers Nestin (green) and SOX2 (magenta). Nuclei were counterstained with DAPI (cyan), and merged images are shown to illustrate co-expression of the stem cell-associated markers. Expression of Nestin and SOX2 was detected in cultures established using both dissociation methods, supporting maintenance of a neural stem/progenitor-associated phenotype following neurosphere generation. Representative images from three independent biological replicates are shown for each condition. Scale bar: 50 μm.

**Figure 6:**
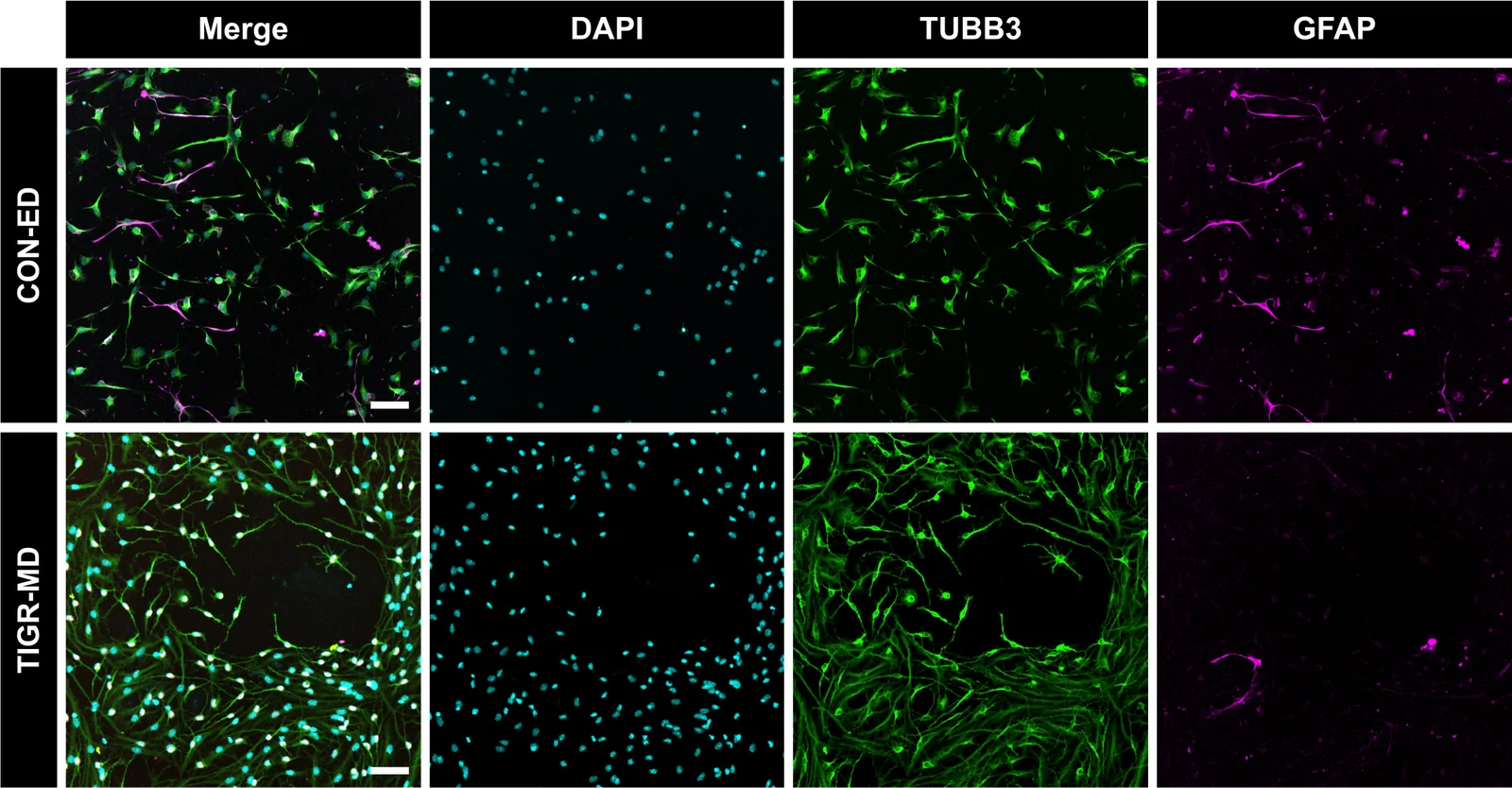
Differentiation of murine adult neural stem cells into neuronal and astrocytic lineages Representative immunofluorescence images of differentiated cultures established using CON-ED or TIGR-MD. Cells were stained for the neuronal marker TUBB3 (green) and the astrocytic marker GFAP (magenta), with nuclei counterstained using DAPI (cyan). TUBB3-positive neuronal and GFAP-positive astrocytic cells were detected in cultures established using both dissociation methods. Representative images from three independent biological replicates are shown for each condition. Scale bar: 50 μm.

Some variability in neurosphere yield and growth kinetics between preparations is expected and may result from differences in microdissection accuracy, tissue processing time, animal age, and dissociation efficiency. Optimization of tissue handling and dissociation parameters may therefore be required depending on the experimental context.

## Quantification and statistical analysis

Neurospheres were quantified on day 12 in cultures established using TIGR-MD or CON-ED. Each sample represented a neurosphere culture established from one mouse brain and was treated as one independent biological replicate. One predefined 4× field of view was analyzed per well (*n* = 6 per group). TIGR-MD cultures contained 17.0 ± 3.85 neurospheres per field of view, whereas CON-ED cultures contained 5.5 ± 1.38 neurospheres per field of view, corresponding to an approximately 3.1-fold increase in TIGR-MD cultures. Data are presented as individual values with mean ± SD in Figure 7; error bars represent the sample standard deviation. Differences between groups were analyzed using a two-tailed unpaired Welch’s *t* test (p = 0.0004).

**Figure 7:**
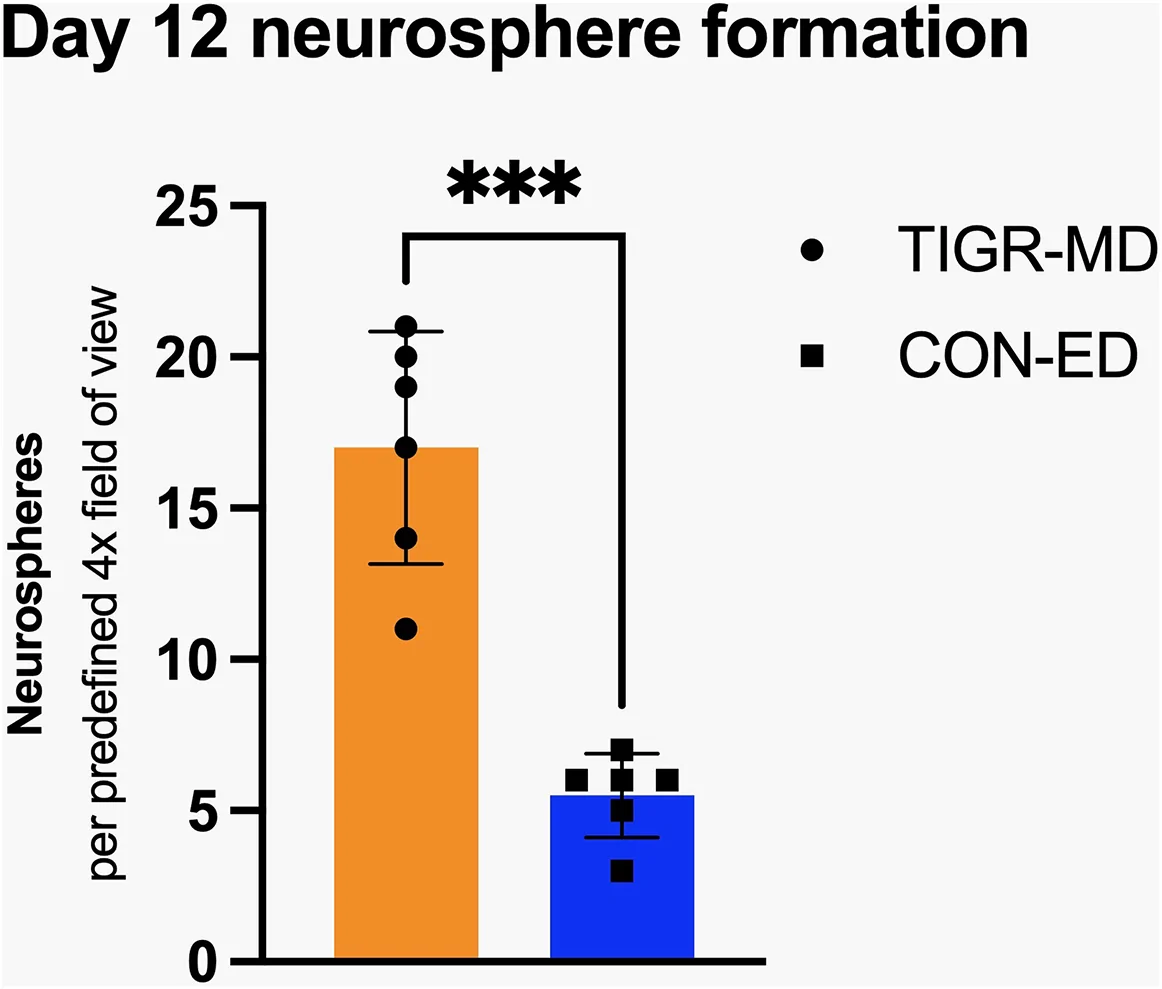
Day 12 neurosphere formation following TIGR-MD and CON-ED Neurospheres were quantified on day 12 in one predefined 4× field of view per well in cultures established using TIGR-MD or CON-ED. Each data point represents one independent biological replicate derived from one mouse (n = 6 per group). Data are presented as individual values with mean ± SD; error bars represent the sample standard deviation. Statistical significance was assessed using a two-tailed unpaired Welch’s *t* test. The exact p value was 0.0004. ∗∗∗ indicates p < 0.001.

In addition, live and dead cell concentrations were determined after splitting TIGR-MD- and CON-ED-derived neurosphere cultures at passage 10 using trypan blue exclusion and manual counting with a hemocytometer (Figures 8 and 9). Each sample represented a culture established from one mouse brain and was treated as one independent biological replicate (*n* = 4 per group). TIGR-MD cultures had a mean live cell concentration of 5.216 × 10^6^ cells/mL, compared with 4.025 × 10^6^cells/mL for CON-ED cultures. Mean dead cell concentrations were 0.1375 × 10^6^ cells/mL for TIGR-MD and 0.2500 × 10^6^ cells/mL for CON-ED. Differences between groups were analyzed using two-tailed unpaired Student’s *t*-tests because no significant differences in variance were detected. Exact *p* values were 0.0382 for live cell concentration and 0.0045 for dead cell concentration (Figure 9).

**Figure 8:**
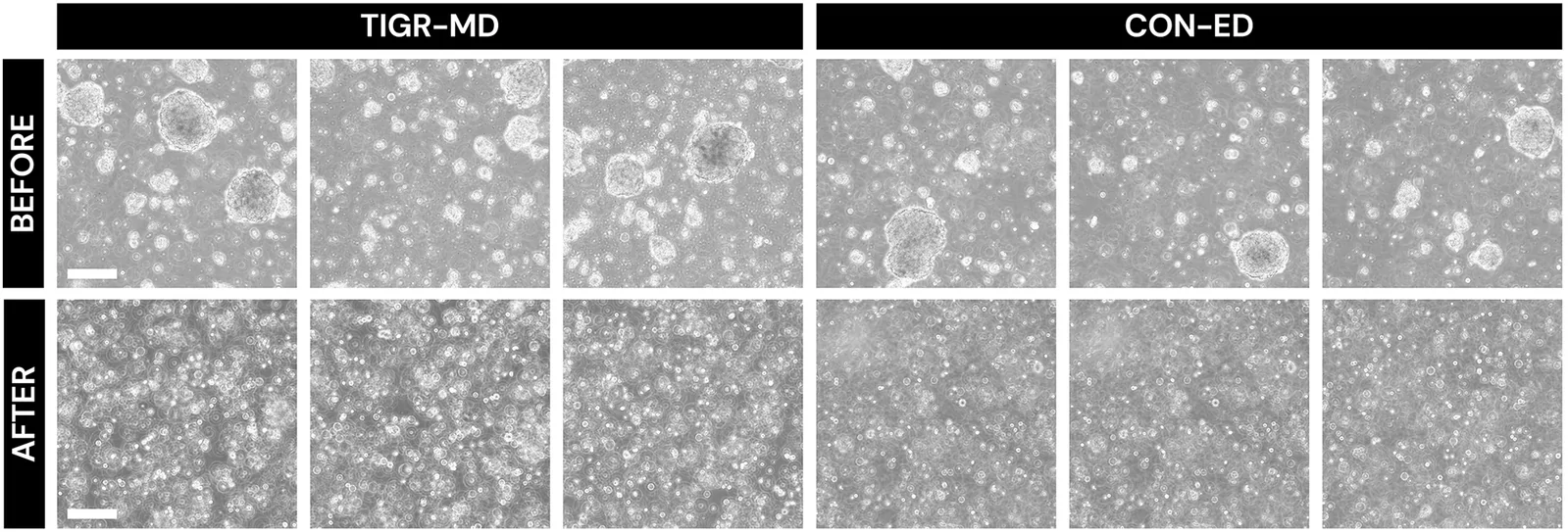
Morphology of TIGR-MD- and CON-ED-derived cultures before and after splitting at passage 10 Representative bright-field images show established neurospheres immediately before splitting and the resulting dissociated cell suspensions after splitting at passage 10. Images are shown from three independent biological replicates, each derived from one mouse brain. Scale bar: 200 μm.

**Figure 9:**
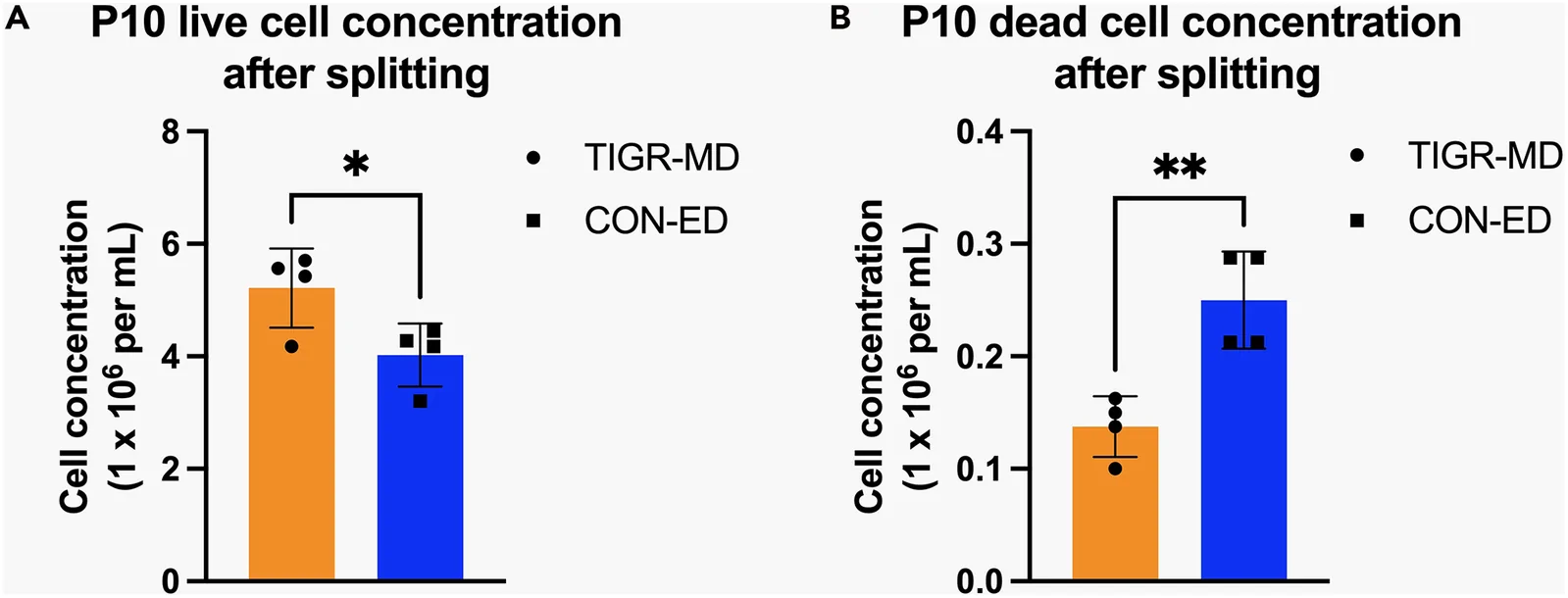
Live and dead cell concentrations after passaging at passage 10 Live and dead cell concentrations were determined after dissociation of TIGR-MD- and CON-ED-derived neurosphere cultures at passage 10 using trypan blue exclusion and manual counting with a hemocytometer. (A) TIGR-MD cultures showed a higher live cell concentration than CON-ED cultures. (B) TIGR-MD cultures showed a lower dead cell concentration than CON-ED cultures. Each data point represents one independent biological replicate derived from one mouse brain (*n* = 4 per group). Cell concentrations are expressed as ×10^6^ cells/mL. Data are presented as individual values with mean ± SD. Statistical significance was assessed using a two-tailed unpaired Student’s *t* test. Exact *p* values were 0.0382 for live cell concentration and 0.0045 for dead cell concentration. ∗ indicates *p* < 0.05; ∗∗ indicates *p* < 0.01.

The relative proportion of dead cells was calculated by dividing the number of trypan blue-positive cells by the total number of counted cells and multiplying by 100. TIGR-MD-derived cultures contained 2.70% ± 0.75% dead cells, whereas CON-ED-derived cultures contained 6.23% ± 0.75% dead cells at passage 10, corresponding to an approximately 57% lower relative proportion of dead cells in TIGR-MD cultures. The relative dead cell proportions were compared using a two-tailed unpaired Student’s *t* test (*p* < 0.001).

All statistical analyses were performed using GraphPad Prism version 11.0.2 (100). Data are presented as individual values with mean ± SD. Statistical significance was defined as *p* < 0.05. In the figures, ∗ indicates *p* < 0.05, ∗∗ indicates *p* < 0.01, ∗∗∗ indicates *p* < 0.001.

## Limitations

This protocol was developed to improve the initial dissociation of adult murine neurogenic tissue and the establishment of primary neural stem/progenitor-enriched neurosphere cultures. The term *enzyme-free* applies specifically to the initial dissociation of primary brain tissue using TIGR-based mechanical dissociation (TIGR-MD). Established neurosphere cultures are subsequently passaged using trypsin-EDTA. Therefore, the complete culture workflow should not be considered entirely enzyme-free. Alternative reagents for passaging, such as accutase, were not evaluated in this study.

The protocol was established using pooled dentate gyrus (DG) and subventricular zone (SVZ) tissue from one adult C57BL/6 mouse per preparation. Separate region-specific cultures were not established or independently evaluated. Consequently, all representative images and quantitative outcomes reflect pooled DG-SVZ cultures and should not be interpreted as independent validation of cultures derived from either region. Adaptation may be required for other brain regions, animal ages, mouse strains, species, or tissue quantities. Higher tissue loads within a single TIGR tube were not systematically evaluated.

Adult neural stem/progenitor cells are relatively rare, and successful culture establishment remains sensitive to microdissection accuracy, tissue-processing time, animal-to-animal variation, and operator-dependent handling. Variability in neurosphere number and growth kinetics between preparations should therefore be expected. The present study did not establish a validated total primary-cell yield or a minimum numerical seeding density per adult brain.

The day-12 quantitative analysis was based on one predefined 4× field of view per well and six independent biological replicates per condition. It therefore provides a comparative estimate of neurosphere formation under the tested conditions rather than a whole-well neurosphere count, sphere-size distribution, or denominator-based neurosphere-forming efficiency.

The passage-10 trypan-blue analysis measured live- and dead-cell concentrations after splitting established cultures. These measurements do not directly assess cell viability or total cell recovery immediately after the initial TIGR-MD or CON-ED dissociation step. Conclusions concerning the initial preservation of cell viability should therefore be avoided. Although cultures established using both methods were maintained for at least 20 passages, systematic passage-wise growth kinetics were not determined.

Neurosphere morphology alone does not confirm adult neural stem/progenitor identity. Expression of Nestin and SOX2 supports maintenance of a neural stem/progenitor-associated phenotype, while differentiation into TUBB3-positive neuronal and GFAP-positive astrocytic cells demonstrates neuronal and astrocytic differentiation potential under the conditions tested. Formal clonal self-renewal assays and differentiation into oligodendrocyte-lineage cells were not performed. Therefore, full self-renewal capacity and trilineage multipotency were not established, and application-specific validation is required before these cultures are used in further functional studies.

## Troubleshooting

### Problem 1: Reduced cell viability due to prolonged tissue handling

Harsh pipetting, rapid trituration, repeated resuspension, or air bubble formation can shear fragile adult neural stem/progenitor cells and neurospheres, resulting in reduced viability, increased debris, low cell recovery, or impaired neurosphere formation. This can occur during CON-ED trituration, pellet resuspension after TIGR-MD or washing, and neurosphere passaging.

**Related to:** CON-ED step 8g; TIGR-MD step 9d; post-dissociation washing and resuspension steps 10–11; passaging steps 16–20.

### Potential solution

• Pipette slowly and consistently to minimize shear stress.
• Keep the pipette tip below the liquid surface to avoid introducing air bubbles.
• Avoid excessive trituration once the suspension appears sufficiently homogeneous.
• Use wide-bore or cut pipette tips when handling fragile cell aggregates or neurospheres.
• Resuspend pellets gently in aNSC growth medium and avoid repeated pipetting to dryness.
• During passaging, limit enzymatic exposure and trituration to the minimum required to obtain a single-cell or small-clump suspension.

### Problem 2: Low cell yield due to inaccurate microdissection

Murine aNSCs are enriched in defined neurogenic regions, and inaccurate isolation of the DG or SVZ can reduce the number of recoverable stem/progenitor cells. Poor anatomical identification may result in low neurosphere numbers even when dissociation and culture conditions are technically correct.

**Related to:** Dissection steps 4–7.

### Potential solution

• Carefully identify coronal sections containing the DG and SVZ before tissue collection.
• Use a clean scalpel and fine dissection tools to minimize inclusion of non-target tissue.
• Keep tissue submerged in ice-cold dissection buffer during region isolation to prevent drying.
• Practice microdissection on training tissue before processing experimental samples.

### Problem 3: High cell mortality due to prolonged enzymatic digestion

Excessive exposure to trypsin-EDTA can compromise membrane integrity and reduce cell viability. This problem is specific to CON-ED and can result in increased debris, reduced cell recovery, and impaired neurosphere formation.

**Related to:** CON-ED steps 8b-f.

### Potential solution

• Begin checking tissue dissociation after 15 min of trypsin-EDTA incubation.
• Do not exceed 30 min total digestion time.
• Use gentle orbital shaking to distribute the enzyme evenly without adding excessive mechanical stress.
• Neutralize enzymatic activity promptly with trypsin inhibitor once sufficient dissociation is observed.
• Avoid delays between enzymatic digestion, inhibition, and washing.

### Problem 4: Incomplete tissue dissociation or persistent clumping

Incomplete dissociation can trap cells in tissue fragments, reduce cell recovery, and interfere with even seeding. In CON-ED, this may result from insufficient enzymatic digestion or inadequate trituration. In TIGR-MD, persistent clumping may occur if tissue fragments are too large or are not positioned correctly at the rotor-stator interface.

**Related to:** Dissection step 6; CON-ED steps 8; TIGR-MD step 9a.

### Potential solution

• Mince tissue into small fragments before dissociation.
• For TIGR-MD, ensure that tissue is positioned correctly at the rotor-stator interface and that the “Soft” program runs for the complete cycle.
• For CON-ED, confirm that trypsin-EDTA is active and within its expiration date.
• Triturate with controlled pipetting, starting with a P1000 and switching to a P200 only for finer dissociation.
• For CON-ED, pass the suspension through a 100 μm cell strainer before seeding if residual clumps remain.

### Problem 5: Low cell yield due to pellet loss during centrifugation or washing

Cell pellets after murine aNSC isolation may be small, loose, or difficult to visualize. Accidental aspiration of the pellet during supernatant removal can substantially reduce cell yield and impair culture establishment. This is especially relevant after TIGR-MD centrifugation and during CON-ED pelleting and washing steps.

**Related to:** CON-ED step 8a and post-dissociation washing steps 10–11; TIGR-MD steps 9b-c.

### Potential solution

• Confirm that centrifugation conditions match the protocol.
• For TIGR-MD, centrifuge at 200 × *g* for 3 min at 24°C.
• For CON-ED, use the centrifugation conditions specified for tissue collection and washing.
• Orient tubes consistently in the centrifuge so that the pellet forms in a predictable position.
• Aspirate the supernatant slowly and leave a small residual volume above the pellet.
• Avoid unnecessary tube transfers and resuspend pellets gently.

### Problem 6: Poor neurosphere formation after seeding

Poor neurosphere formation may result from low starting cell number, reduced viability, excessive debris, incorrect culture vessel type, insufficient growth factor activity, or disruption during early culture establishment.

**Related to:** Cell seeding and initial culture steps 12–14.

### Potential solution

• Keep cells in fresh aNSC growth medium containing active EGF and bFGF.
• Use ULA plates to prevent premature cell adhesion.
• Avoid disturbing cultures during the first 48 h after seeding.
• During medium changes, aspirate gently from the well edge and replace only half of the medium.
• If neurosphere formation remains poor, review dissection accuracy, tissue handling time, dissociation efficiency, and animal age.

### Problem 7: Poor recovery after passaging

Poor recovery after passaging may result from excessive trypsin-EDTA exposure, harsh trituration, incomplete neutralization of enzymatic activity, or unsuitable reseeding density. This can delay neurosphere reformation and increase debris accumulation after passage.

**Related to:** Passaging steps 15–22.

### Potential solution

• Passage neurospheres when they reach approximately 50–100 μm in diameter.
• Limit trypsin-EDTA exposure during passaging to the indicated 2 min.
• Add trypsin inhibitor promptly to stop enzymatic activity.
• Triturate gently and avoid over-dissociation once a sufficient single-cell or small-clump suspension is obtained.
• Reseed cells in fresh aNSC growth medium and maintain cultures in ULA vessels.

## Resource availability

### Lead contact

Further information and requests for resources and reagents should be directed to and will be fulfilled by the lead contact, Lisa Sevenich (lisa.sevenich@med.uni-tuebingen.de).

### Technical contact

Technical questions on executing this protocol should be directed to and will be answered by the technical contacts, Vanessa Arnold (vanessa.arnold@med.uni-tuebingen.de) and Anna Wolfram (anna.wolfram@uni-tuebingen.de).

### Materials availability

This study did not generate new unique reagents. There are restrictions to the availability of mice due to animal welfare restrictions.

### Data and code availability

This study did not generate original code. Source data supporting the findings of this study, including the underlying Excel files, are available from the lead contact upon reasonable request.

## Acknowledgments

This project was supported by institutional funds from the Department of Neurology and Interdisciplinary Neuro-Oncology and the M3 Research Center; the Hector Foundation MINT Personal Fonds; and the 10.13039/501100001659Deutsche Forschungsgemeinschaft (DFG) Cluster of Excellence EXC2180 “Image-Guided and Functionally Instructed Tumor Therapies” (iFIT). Additional support was provided by the 10.13039/100008662Joachim Herz Stiftung through Add-on Fellowships awarded to V.A. and A.W. and a Kickstart Fund awarded to A.W., as well as by the 10.13039/501100005846Friedrich-Ebert-Stiftung, awarded to A.W. and V.A. The TIGR Tissue Grinder and Dissociator was provided by OMNI Life Science GmbH & Co. KG.

## Author contributions

Conceptualization, V.A. and A.W.; methodology, V.A. and A.W.; investigation, V.A., A.W., and A.M.; data curation, V.A., A.W., V.S.-K., A.S., and S.A.; visualization, V.A., A.W., and A.M.; resources, L.S.; supervision, V.A., A.W., and L.S.; project administration, A.W.; funding acquisition, L.S. and A.W.; writing – original draft, V.A. and A.W.; writing – review and editing, V.A., A.W., and L.S.

## Declaration of interests

The authors declare no competing interests.
